# DXA-Measured Total and Regional Body Composition in Female Athletes with a Physical Impairment

**DOI:** 10.3390/jfmk10010049

**Published:** 2025-01-28

**Authors:** Valentina Cavedon, Ilaria Peluso, Elisabetta Toti, Marco Sandri, Anna Pedrinolla, Carlo Zancanaro, Chiara Milanese

**Affiliations:** 1 Department of Neurosciences, Biomedicine and Movement Sciences, University of Verona, 37129 Verona, Italy; carlo.zancanaro@univr.it (C.Z.); chiara.milanese@univr.it (C.M.); 2 Research Centre for Food and Nutrition (CREA-AN), 00178 Rome, Italy; ilaria.peluso@crea.gov.it (I.P.); elisabetta.toti@crea.gov.it (E.T.); 3 Big & Open Data Innovation Laboratory (BODaI-Lab), University of Brescia, 25123 Brescia, Italy; sandri.marco@gmail.com; 4 Department of Cellular, Computational and Integrative Biology—CIBIO, University of Trento, 38123 Trento, Italy; anna.pedrinolla@unitn.it

**Keywords:** paralympic athletes, fat mass, lean mass, bone mineral content, spinal cord injury, amputation, low energy availability

## Abstract

**Background/Objectives:** In recent years, awareness has been growing regarding the needs of female athletes with physical impairments. Despite the importance from both health and performance perspectives of assessing body composition in this athletic population, there is limited literature focusing on this topic. This study explored whole-body and regional three-compartment body composition in female athletes with a physical impairment to assess the impact of impairment and sex on body composition parameters in this population. **Methods:** Twenty female athletes with a physical impairment were pair-matched by age with an able-bodied female athlete and a male athlete with a comparable physical impairment. All athletes underwent whole-body scanning with dual-energy X-ray absorptiometry. **Results:** Female athletes with physical impairments showed body composition changes including higher amounts of fat mass, particularly in the lower body regions. Among athletes with a physical impairment, sex showed an independent effect on whole-body composition, with females showing higher fat mass and lower lean mass and bone mineral content compared with males, especially in the legs. **Conclusions:** Female athletes with physical impairments had a distinct body composition profile, characterized by sex-specific distribution of body tissue at the regional level. Nutritional and training strategies aimed at optimizing body composition in female athletes with physical impairments should be specifically tailored to meet the needs of this athletic population.

## 1. Introduction

In the sports field, assessing an athlete’s body composition provides insight into the relative proportion of fat mass, lean mass, and other essential components. This assessment serves as a valuable tool for professionals working with athletes, as it helps in understanding the athlete’s nutritional status and monitoring the combined effects of dietary intake and training [1]. When interpreting an athlete’s body composition, it is important to recognize that it reflects a complex interaction of multiple interconnected factors, including genetic predisposition, age, sex, race, ethnicity, medication, health conditions, and lifestyle choices such as diet and physical activity levels [2]. Furthermore, in the case of athletes, the sports science literature has long demonstrated that body composition is also influenced by the type of sport practiced, the athlete’s specific role or playing position (especially in team sports), training volume, and level of athletic performance [1].

These interactions among interconnected factors become even more complex in Paralympic athletes, such as wheelchair basketball players, sitting volleyball players, and Para skiers, as well as in athletes participating in adapted sports not yet part of the Paralympic Games, like amputee soccer or Para climbing. In fact, in this unique athletic population, body composition is influenced by additional factors, including the type of impairment (e.g., spinal cord injury, limb amputation, multiple sclerosis), impairment severity (e.g., level of spinal cord injury or amputation), duration of the injury, and the specific adapted sport practiced [3,4,5,6,7].

In recent years, the scientific community has increasingly focused on various aspects of body composition in athletes with physical impairments, including the validity of techniques used to estimate the different compartments of body composition, including efforts to provide reference data for characterizing this population and monitoring changes throughout the competitive season [3,4,5,6,7,8,9,10,11,12,13]. Despite the importance of assessing body composition in athletes with physical impairments [7] and the growing participation of female athletes in both Paralympic and other international events [14], research in this area is still in its early stages compared with studies on able-bodied athletes. To date, research in this field has predominantly focused on male athletes with physical impairments or on male and female athletes as a combined group, leaving a notable lack of female-specific investigations. This research gap might lead to inappropriate extrapolation of findings from male athletes with physical impairments to female athletes, or from able-bodied female athletes to those with a physical impairment. Therefore, there is a need for scientific data on the body composition of female athletes with a physical impairment to better characterize this unique athletic population.

In this study, we assessed body composition in female athletes with a physical impairment using dual-energy X-Ray absorptiometry (DXA), considered the gold standard for evaluating body composition in this athletic population [15]. The first aim of this study was to explore the impact of physical impairment on body composition by comparing female athletes with a physical impairment to an age- and sex-matched control group of able-bodied female athletes. The second aim of this study was to investigate sex-related differences in body composition by comparing DXA-measured variables of female athletes with a physical impairment against those of their male counterparts, matched for type and severity of physical impairment. We hypothesized that female athletes with a physical impairment would exhibit a distinct body composition phenotype, differing both from able-bodied female athletes and from their male counterparts.

## 2. Materials and Methods

For the study sample, the inclusion criteria were age > 18 years, participation in an adapted sport at a competitive level, and at least one season of sport practice. The exclusion criteria included pregnancy or breastfeeding, or the presence of other comorbidities that could affect body composition (e.g., diabetes) in addition to the disability. Twenty female athletes with a physical impairment, aged 34.4 ± 8.2 years, met the inclusion and exclusion criteria and volunteered to participate in this cross-sectional study.

Physical impairments included spinal cord injury at the cervical (n = 1), thoracic (n = 2), and lumbar level (n = 1), monoplegia (n = 2), hemiplegia (n = 1), ataxia (n = 1), cerebral palsy (n = 1), nerve damage (n = 2), unilateral transfemoral amputation (n = 2), bilateral transfemoral amputation (n = 1), partial upper limb amelia (n = 2), and other musculoskeletal conditions such as reduced range of movement and limb deformities (n = 4). The origin of the disability was acquired (n = 14) or congenital (n = 6). Among those with acquired disabilities, the causes were traumatic (n = 13) or due to illness (n = 1). The average duration of the injury was 14.4 ± 9.7 years, and the average time since starting (or restarting) their athletic career after injury was 6.9 ± 8.4 years. The female athletes had practiced adapted sports (e.g., sitting volleyball, wheelchair basketball, Para table tennis, wheelchair fencing, wheelchair Rugby, Para alpine skiing, and Para climbing) for at least one competitive season. The average experience in the practiced adapted sport was 4.6 ± 5.3 years, and the average amount of training was 5.6 ± 1.9 h per week. Female athletes with a physical impairment were either wheelchair-dependent (n = 3) or able to ambulate, some with the use of support devices such as crutches or prostheses (n = 17).

According to the purposes of this study, each female athlete with a physical impairment was matched 1:1 with an able-bodied female athlete of similar age (±2 years) and a male athlete with a similar physical impairment. Matching criteria for both control groups included practicing a sport (or an adapted sport in the case of male athletes with physical impairments) at a competitive level for at least one year. Female non-disabled athletes were involved in various sports such as track and field, alpine skiing, swimming, cycling, dancing, and karate, while male athletes with physical impairments participated in adapted sports like wheelchair basketball, wheelchair rugby, Para table tennis, sitting volleyball, amputee soccer, and handcycling. To match female athletes with a physical impairment to their male counterparts, the criteria included selecting a male athlete with a similar impairment in terms of type (i.e., neurological or musculoskeletal condition), location of impairment (i.e., upper limb(s), lower limb(s), or both), and ambulation modality in daily life (i.e., wheelchair-dependent or non-wheelchair-dependent). For the type of impairment, we applied strict matching criteria, pairing athletes with similar subtypes (e.g., spinal cord injury, limb amputation) and severity (e.g., level of lesion or amputation) where possible.

### 2.1. Testing Procedures

Testing took place in the late morning or early afternoon, after a 3–4 h fast. All participants were instructed to avoid strenuous physical activity the day before each measurement session and to refrain from exercising on the day of the measurements. Before the DXA scan, a questionnaire was administered to the participants to confirm their eligibility and gather general information to characterize the sample. Body mass and stature, required by the DXA software (Hologic APEX Software, version 5.6.1.3) for scanning, were assessed as follows: for athletes who were able to stand, body mass was measured to the nearest 0.1 kg using an electronic scale (Tanita BWB-800 MA, Wunder SA.BI. Srl, Milano, Italy), and stature was measured to the nearest 0.5 cm with a Harpenden stadiometer (Holtain Ltd., Crymych, Pembrokeshire, UK), following conventional criteria and procedures [16]. For athletes who were wheelchair users and unable to stand, body mass and stature were self-reported. Body mass index for all participants was calculated as body mass (kg) divided by height squared (m^2^).

Body composition was assessed using a DXA total body scanner (QDR Horizon, Hologic, MA, USA; fan-beam technology). The assessment followed “The Best Practice Protocol for the Assessment of Whole-Body Body Composition by DXA” [17], with slight adaptations to accommodate participants with physical impairments, as described by Cavedon et al. [3,7]. Specifically, athletes were instructed to void their bladder and remove all metal, jewelry, or reflective materials, including prostheses, if possible, before the DXA scan. Additionally, athletes were required to wear only underwear during scanning.

### 2.2. Data Analysis and DXA Outcomes

Analysis of the scans was performed by one trained researcher. Specific anatomical landmarks were used to delineate the standard regions of interest (trunk, arms [right and left], legs [right and left]). The android and gynoid regions were defined automatically by the software. For statistical purposes in this study, the left and right arms, as well as the left and right legs, were considered as one region each (i.e., arms and legs, respectively).

At the whole-body level, the following DXA variables were considered: total mass (expressed in grams), lean mass (expressed in grams), bone mineral content (expressed in grams), fat mass (expressed in grams), relative fat mass (expressed as a percentage), and the fat-to-lean mass ratio. At the regional level (i.e., in the arms, trunk, and legs), we analysed the DXA values for fat mass, lean mass, bone mineral content, total mass, and relative fat mass. For the android and gynoid regions, only fat mass (expressed in absolute terms), relative fat mass, and the android-to-gynoid ratio were included in the analysis. Additionally, the appendicular lean mass index was calculated as the ratio of appendicular lean mass (i.e., the sum of lean mass in the arms and legs, expressed in kilograms) to the square of height (in meters).

### 2.3. Statistical Analysis

Descriptive statistics (mean and standard deviation) were computed for all variables. Data normality was assessed using the Kolmogorov–Smirnov test, and variance homogeneity was evaluated with Levene’s test. When necessary, data were transformed using the method described by Box and Cox [18], and parametric tests were applied.

A two-tailed Student’s *t*-test for independent samples was used to compare means between two groups. Cohen’s d was calculated to assess effect size in the Student’s *t*-test for independent samples, with effect sizes interpreted as small (d = 0.2), medium (d = 0.5), or large (d = 0.8), following Cohen’s guidelines [19].

All analyses were performed using SPSS v.16.0 (IBM Corp., Armonk, NY, USA). Statistical significance was set at *p* value ≤ 0.05.

## 3. Results

Descriptive statistics for all the variables considered are presented in Table 1.

The *t*-test showed no statistically significant differences in age between groups (*p* > 0.05 for all comparisons; Table 2). The group of females with a physical impairment had an average weight of 59.8 ± 14.1 kg and stature of 161.8 ± 8.2 cm, neither of which were significantly different from the able-bodied female group (55.1 ± 5.6 kg and 164.6 ± 4.7 cm, *p* > 0.05; Table 2). The group of females with a physical impairment had a body mass index of 22.8 ± 5.1 kg/m^2^, which was comparable to the male group with physical impairments (23.6 ± 2.8 kg/m^2^, *p* > 0.05; Table 2). Females with a physical impairment had a statistically significantly higher body mass index (on average −2.5 kg/m^2^) than able-bodied females. They also showed lower body weight (on average −12.9 kg) and shorter stature (on average −13.1 cm) compared with males with a physical impairment (Table 2).

### 3.1. Comparison of DXA-Measured Body Composition Between Females with a Physical Impairment and Able-Bodied Females

**Figure 1:**
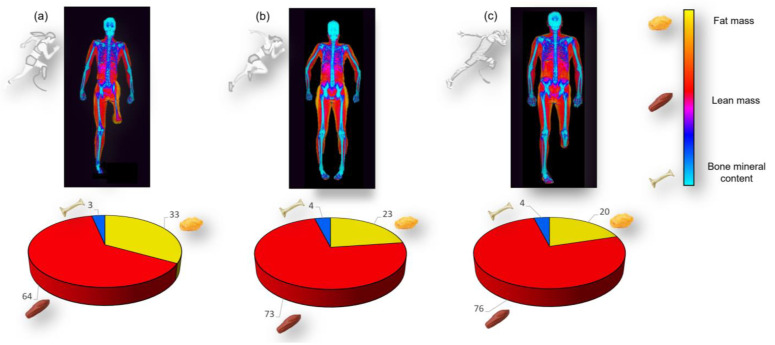
Schematic representation of the results of female athletes with a physical impairment (**a**), female able-bodied athletes (**b**), and male athletes with physical impairments (**c**).

**Figure 2:**
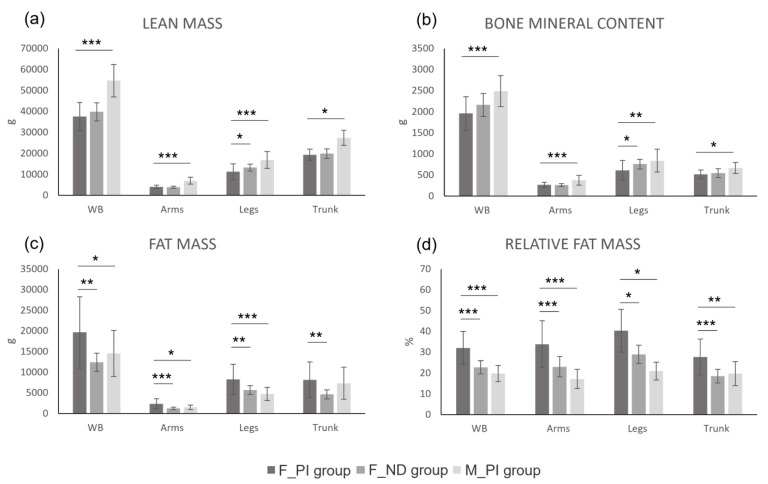
Graphical representation of the results of the independent samples *t*-test. Lean mass (**a**), bone mineral content (**b**), absolute fat mass (**c**), and relative fat mass (**d**). Legend: WB, whole-body level; F_PI group, female athletes with a physical impairment; F_ND group, able-bodied female athletes; M_PI group, male athletes with a physical impairment. *, *p* < 0.05; **, *p* < 0.01; ***, *p* < 0.001.

As reported in Table 2 and in Figure 1 and Figure 2, the *t*-test showed that, at the whole-body level, the group of females with a physical impairment had higher fat mass (both absolute and relative) and a higher fat-to-lean mass ratio compared with the group of able-bodied females. At the whole-body level, no statistically significant differences were found between the two groups for lean mass, bone mineral content, or appendicular lean mass index.

**Figure 3:**
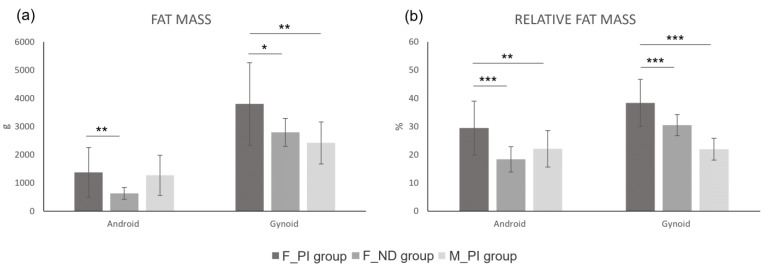
Graphical representation of the results of the independent samples *t*-test in the android and gynoid regions. Absolute fat mass (**a**) and relative fat mass (**b**). Legend: F_PI group, female athletes with a physical impairment; F_ND group, able-bodied female athletes; M_PI group, male athletes with a physical impairment. *, *p* < 0.05; **, *p* < 0.01; ***, *p* < 0.001.

Regionally, statistically significant differences in body composition between female athletes with a physical impairment and able-bodied females were found for fat mass (both absolute and relative) in the arms and trunk regions, with higher values observed in the group of females with a physical impairment compared with their female counterparts (Table 2 and Figure 2). The group of females with a physical impairment also showed higher absolute and relative fat mass in the android (746.8 g and 11.1%, respectively) and gynoid (1003 g and 7.9%, respectively) regions compared with the group of able-bodied females (Figure 3). Females with physical impairments also had a statistically significant higher android-to-gynoid ratio than their able-bodied counterparts (*p* < 0.001). The group of females with a physical impairment displayed statistically significant higher fat mass (both absolute and relative) in the legs, along with lower lean mass and bone mineral content (Table 2 and Figure 2).

### 3.2. Comparison of DXA-Measured Body Composition Between the F_PI Group and the M_PI Group

The *t*-test showed that at the whole-body level, the group of females with a physical impairment had statistically significant higher values for absolute fat mass (about +5 kg on average), relative fat mass (about +12.3% on average), and fat-to-lean mass ratio (about +0.3 points on average), along with statistically significant lower values for total mass (about −12 kg on average), lean mass (about −17 kg on average), bone mineral content (about −0.5 kg on average), and appendicular lean mass index (about −1.9 points on average). Similar differences were observed at the regional level, with the exception of total mass in the leg region and absolute fat mass in both the trunk and android regions (Table 2; Figure 1, Figure 2 and Figure 3).

## 4. Discussion

This study assessed the impact of physical impairment and sex on three-compartment body composition in female athletes with a physical impairment. The present study is the first to investigate body composition in a relatively large cohort of female athletes with a physical impairment in comparison with two control groups matched in a 1:1 ratio: equivalent samples of female able-bodied athletes and of male athletes with a physical impairment. The study group was carefully matched with each control group for key confounding variables. This is crucial in body composition research involving athletes with physical impairment, as the inherent large variability within this population may hamper accurate and reliable findings.

A primary aim of this study was to explore the impact of the impairment on body composition in female athletes with a physical impairment by assessing differences with able-bodied female athletes matched pairwise by age. The results showed that at the whole-body level, female athletes with a physical impairment had higher amounts of fat mass, both absolute and relative, along with higher fat-to-lean mass ratio (Table 2). This difference in relative fat mass between females with a physical impairment and their able-bodied counterparts at the whole-body level (9% on the average) was close to being maintained in the arms (10.8%), trunk (9.1%), and legs (11.4%). These findings are supported by data from Sutton and colleagues [6] showing differences in the distribution of bone, lean, and fat mass at the regional level in a sample of female wheelchair athletes. At partial variance with our results, Sutton and colleagues [6] found improved body composition (i.e., lower relative fat mass and higher amounts of lean mass and bone mineral density) in the arms of female wheelchair athletes vs. able-bodied athletes. This discrepancy was probably due to the limited number of wheelchair athletes in our sample. Future research should address the combined effects of the type of physical impairment and the type of sport practiced in athletes with physical impairments.

The region with the greatest differences in body composition between female athletes with a physical impairment and their able-bodied counterparts was the legs. Here, female athletes with a physical impairment had higher fat mass, lower lean mass, and lower bone mineral content compared with able-bodied female athletes (Table 2; Figure 1a–c). This finding is in accordance with previous data [6] reporting that female wheelchair athletes had significantly lower lean mass and bone mineral density as well as higher fat mass in the legs compared with their able-bodied reference group.

The alterations in body composition due to disability observed in female athletes with a physical impairment, characterized by increased fat mass and reduced lean mass, as well as the pattern of regional body tissue distribution, may have potential health implications. Muscle mass is considered a protective factor against cardiometabolic risk, as it functions as an endocrine organ capable of producing and releasing myokines. These myokines circulate in the bloodstream and help to reduce inflammation and improve insulin resistance [20]. Conversely, an increase in fat mass is strongly linked to metabolic syndrome and insulin resistance, primarily due to its role in triggering proinflammatory processes and oxidative stress [21]. Additionally, the loss of muscle mass can lead to a decrease in basal metabolic rate and a reduction in physical activity levels, which may result in fat mass accumulation even without changes in overall body weight [22].

In the literature regarding able-bodied subjects, it has been shown [23] that regional fat-to-muscle mass ratio is strongly and positively associated with the incidence and mortality risk of cardiovascular diseases, independent of overall obesity. Specifically, in able-bodied females, the fat-to-muscle mass ratio in the legs was reported to have the strongest associations with cardiovascular risk among regional body parts [23]. The significant interplay between fat and lean mass in individuals with spinal cord injury has also been examined [24]. Data have shown that fat-to-lean mass ratio correlates with both high-sensitivity C-reactive protein and interleukin-6 and is considered the strongest predictor of metabolic syndrome [24]. These findings underscore the importance of nutritional strategies aimed at preserving lean mass in female athletes with physical impairments, particularly those with a spinal cord injury.

Of note, the reduction in bone mineral content reported in our study and bone mineral density (reported in [6]) in the legs of female athletes with physical impairments might represent a warning of osteopenia and osteoporosis later in age. In fact, it is known that postmenopausal females tend to lose bone mineral content and bone mineral density [25], with an increased risk of osteoporotic fractures [25]. In addition to that, it is reasonable to assume that female athletes with physical impairments may be at even higher risk of osteoporotic fractures than the general female population because of the negative effects of their physical impairment, e.g. long periods of immobilization and decreased gravitational loading from wheelchair use [3]. Accordingly, training and nutrition for female athletes with physical impairments should be optimized to prevent osteoporosis and the consequent risk of fractures [26]. Furthermore, the literature has frequently reported inadequate vitamin D status in athletes with physical impairments, particularly those with a spinal cord injury. In this context, periodic screening of vitamin D levels in female athletes with physical impairments would be valuable for assessing the need for proactive preventive measures, as well as for providing dietary advice and supplementary prescriptions for bone-supporting nutrients in cases of inadequate vitamin D status, in order to mitigate the risk of bone loss or fractures.

A second aim of this study was to assess the impact of sex on body composition in athletes with a physical impairment. The results supported the experimental hypothesis, showing that female athletes with a physical impairment exhibited a distinct body composition profile compared with their male counterparts. Specifically, females, on average, tended to have higher relative fat mass and lower lean mass and bone mineral content than males, both at the whole-body and regional level. At the whole-body level, female athletes with a physical impairment had, on average, 5 kg more absolute fat mass, relative fat mass, and higher fat-to-lean mass ratio than their male counterparts (Table 1; Figure 2c,d). This result is consistent with previous findings on sex differences in the body composition of wheelchair athletes showing that females tend to have higher fat mass than their male counterparts [4,13,27]. Mojtahedi and colleagues [13] considered a sample of 16 wheelchair athletes (i.e., wheelchair basketball and wheelchair fencing; females, n = 8) with spinal cord injuries, reporting that females had, on average, 11% more DXA-measured fat mass than males at the whole-body level. Similarly, Flueck [4] found that the DXA-measured relative fat mass in 20 female athletes engaged in various wheelchair sports was about 12% higher compared with males (n = 49). Findings in athletes with physical impairments are consistent with those in the general population showing that women have a substantially greater mean relative fat mass (about 10%) than men [28,29]. It therefore seems that when accounting for age and type of impairment in athletes with physical impairments, sex differences in body composition should be considered innate and attributable to a combination of biological, reproductive, hormonal, and metabolic factors [28,29,30,31].

The results of the present work showed that the innate sex differences between females and males observed at the whole-body level were generally maintained at the regional level. In fact, as shown in Figure 2c,d, female athletes with a physical impairment had higher absolute and relative fat mass than males in the arms (0.8 kg and 16.7%, respectively), legs (3.6 kg and 19.7%, respectively), trunk (0.8 kg and 7.9%, respectively), android (0.1 kg and 7.3%, respectively), and gynoid (1.4 kg and 16.4%, respectively) regions. These results are in line with those of Flueck [4], who reported that compared with males, female athletes with a physical impairment had higher relative fat mass at the regional level. The largest difference in relative fat mass between the two sexes was found in the legs (21.3%), followed by the arms (15.2%), and trunk (8.1%) [4]. A similar trend was observed in able-bodied non-athletic adults [28,29]. In fact, it has been reported [28,29] that male and female able-bodied non-athletic adults have similar fat mass in the trunk, showing about 10% more fat mass in the arms (a lower difference than that found in athletes with physical impairments in this study) and 30% more fat mass in the legs (a higher difference than that found in athletes with physical impairments in this study). It may be hypothesized that this discrepancy is due to the different response of females and males with physical impairments to training and dietary interventions compared with able-bodied individuals, especially at the regional level.

We have shown herein that females with a physical impairment had higher relative fat mass in the arms than in the trunk region, while the opposite was found in their male counterparts (Figure 1a,c). Furthermore, although both females and males had a higher relative fat mass in the legs compared with the upper body, females exhibited a greater relative fat mass in the legs than males. Females also displayed a lower android-to-gynoid fat ratio than males, consistent with greater fat accumulation in the lower body in females. This pattern of fat mass distribution is consistent with findings in able-bodied individuals [30] and suggests that females and males with a physical impairment store their fat mass differently, which could contribute to sex differences in energy metabolism that are probably due to the typical demands of female reproductive physiology [32,33].

Fat-free mass is among the main predictors of resting metabolic rate in Paralympic athletes [34]. According to previously studies [4,35], we found lower fat-free mass and appendicular lean mass index in female compared with male Paralympic athletes. Appendicular lean mass index is among the criteria for definition of sarcopenic obesity [36], independent of body mass index. In this study, the latter did not differ significantly between female athletes with a physical impairment and their male counterparts. However, in female athletes with a physical impairment, we observed a higher fat-to-lean mass ratio and relative fat mass at the whole-body level, and in the arms, legs, android and in the gynoid regions, compared with both the group of able-bodied females and the group of male athletes with a physical impairment. Regarding central obesity, the android-to-gynoid ratio in female athletes with a physical impairment was higher compared with able-bodied female athletes, and lower than in male athletes with a physical impairment. In this context, it is important to underline that interleukin 6 levels increased 3.35 pg/mL for every unit increase in android-to gynoid fat mass ratio [37].

In recent years, awareness has been growing of the particular needs of female athletes [38,39,40,41], and Paralympic women are recognized as doubly marginalized by the intersection of gender and disability [42]. Paralympic women have reported barriers in their training processes [43] and a higher percentage than among Olympic female athletes reported their performance to be impacted by their menstrual cycle [44]. In particular, neurologic pain and exacerbation of multiple sclerosis symptoms have been reported by some athletes [44]. Although menstrual dysfunction, low energy availability (LEA), and low bone mineral density are common in Paralympic athletes [45] less than 10% of athletes reported awareness of the Female Athlete Triad [46,47]. Additionally, in addressing issues related to relative energy deficiency in sport, 16% of Paralympic athletes reported not having a relaxed relationship with food intake and body weight, and approximately half had periodically attempted to either increase or reduce their body weight [47]. In the literature, it has also been noted that the majority of Paralympic athletes (60%) are not currently receiving follow-up care from a dietician [47]. As reported in this study, female athletes with a physical impairment have a reduced bone mineral content in the legs compared with able-bodied female athletes, and this requires particular nutritional support. Furthermore, special attention should be given to athletes with skeletal disorders such as amputations, as they require more energy owing to compensatory gait.

Information about body composition in female athletes with physical impairments is indeed crucial for understanding the potential risk factors associated with LEA. Body composition data help identify how LEA may impact their fat and lean mass and are essential for monitoring the health consequences of LEA, such as menstrual dysfunction, bone health issues, and muscle loss. Additionally, understanding body composition can enable the development of individualized interventions aimed at optimizing both health and performance. This could involve adjusting nutrition, exercise, and recovery protocols based on the athlete’s specific body composition characteristics, ensuring they are able to meet the energy demands of both training and daily physiological functions. Based on the results of the present study and the available literature on this athletic population, we believe that female athletes with physical impairments require specific nutritional support. This includes education and counseling, assistance with managing menstrual disorders, and periodic health evaluations. Such evaluations should involve a comprehensive and multidisciplinary approach to athlete health, including accurate assessments of body composition and bone health, to evaluate the combined effects of diet and training. The Female Athlete Triad is a crucial concept in female athlete performance and health, particularly for athletes with physical impairments such as spinal cord injuries. Research employing a multidisciplinary approach is essential to support medical staff and assist athletes with physical impairments in preventing health issues associated with low energy availability, menstrual dysfunction, and low bone mineral density.

This study has some limitations that should be acknowledged. First, the relatively limited number of female athletes with a physical impairment in this study prevented the investigation of body composition according to the type of physical impairment (e.g., spinal cord injury or limb amputation/s), as well as analysis of the combined effects of the type and severity of physical impairment and the type of sport practiced. Second, we were unable to assess the dietary habits and basal metabolism of the participants, which could have affected their body composition. Second, we were unable to assess the participants’ dietary habits, basal metabolism, or menstrual cycle, or the presence of menstrual dysfunctions, which could have affected their body composition.

Future research should aim to include a larger and more diverse cohort to enable subgroup analyses and enhance the generalizability of the findings. Specifically, future studies should investigate the combined effects of disability type and severity, type of sport, dietary intake, basal metabolism, and the complexities of female physiology, including the menstrual cycle and the presence of menstrual disfunctions. Investigating menstrual health parameters along with the previously mentioned parameters in future studies would provide a holistic understanding of the unique challenges faced by female athletes with impairments. To this end, longitudinal studies including basal metabolism measurements, dietary intake data, and clinical analyses could more effectively assess changes in body composition over time, as well as the potential causal relationships between training, diet, and body composition.

In this study, we assessed neither sport performance nor physical activity levels in female athletes with a physical impairment. To date, the relationships between body composition, sport performance, and physical activity levels in female athletes with physical impairments have been poorly explored in the scientific literature. However, based on evidence from studies involving male athletes with physical impairments, we can speculate that, as in males, the relationship between body composition and sport practice in females is indeed bilateral, with each influencing the other [7,48].

On one hand, in males, sport practice has been shown to influence body composition by increasing lean mass, reducing fat mass, optimizing bone health, and mitigating the alterations in body composition associated with disability [7]. On the other hand, body composition can significantly influence sport performance. The amount and distribution of muscle and fat affect an athlete’s strength, endurance, and agility, all of which are critical for sport-specific tasks. For instance, in sitting volleyball, studies have shown that a higher relative fat mass at both the subtotal and regional levels is associated with poorer performance in sport-specific sprint tests, particularly those involving changes in direction [48]. In the future, it would be interesting to investigate how regular sport practice influences body composition in female athletes with a physical impairment, considering the type of impairment and the type and volume of sport practiced, to gain a deeper understanding of which body composition parameters can enhance athletic performance in females.

## 5. Conclusions and Practical Perspectives

In conclusion, the results of the present study highlight that female athletes with a physical impairment exhibit a distinct body composition profile compared with female able-bodied athletes and male athletes with a physical impairment, characterized by a sex-specific pattern of body fat and lean tissue distribution at the regional level.

From a methodological point of view, the results of this study underline the importance of assessing body composition in this athletic population using DXA, as it provides body composition measurements at the regional level. These allow nutritionists and clinicians to appreciate the specific distribution patterns of body tissues and the regional variations in body composition that may result from the combined effects of diet and sport training. As DXA is often inaccessible in many clinical and sports settings due to logistical constraints and costs, professionals working with female athletes with physical impairments should be aware that no female-specific predictive equations are available for field-based methods (e.g., anthropometry or bioelectrical impedance analysis) when DXA is not an option. It is reasonable to hypothesize that predictive equations derived from able-bodied populations or male athletes with a physical impairment may not be suitable for assessing body composition in female athletes with physical impairments. Therefore, DXA should be considered the preferred method, and future research should aim to develop predictive equations specific to female athletes with physical impairments, for use in the field when DXA is unavailable.

In the context of nutritional and training strategies aimed at improving (or maintaining) body composition, the results of the present study underline the importance of tailoring these to the specific body composition phenotype of this athletic population of female athletes with physical impairments. From a clinical perspective, considering the importance of regional fat-to-muscle mass ratio and bone mineral density in female athletes with physical impairments, evaluation of body composition and bone health through DXA should be included in the periodic health evaluations of this population, particularly in the nutritional evaluations of those with spinal cord injuries [49], along with questions about menstrual disturbances, in order to address (and possibly prevent) issues related to relative energy deficiency in sports and its severe consequences [47].

## Figures and Tables

**Table 1:** Descriptive statistics for all the variables considered.

	F_PI		F_ND		M_PI	
	(n = 20)		(n = 20)		(n = 20)	
	Mean	SD	Mean	SD	Mean	SD
**Demographic and anthropometric characteristics**						
Age (years)	34.4	8.5	34.3	8.5	36.5	10.7
Weight (kg)	59.8	14.1	55.1	5.6	72.7	13.3
Stature (cm)	161.8	8.2	164.6	4.7	174.9	9.9
BMI (kg/m^2^)	22.8	5.1	20.3	1.6	23.6	2.8
**Whole-body composition**						
FM (g)	19,645.5	8620.6	12,391.0	2164.6	14,531.1	5552.4
LM (g)	37,623.7	6567.5	39,814.6	4345.7	54,670.4	7736.5
BMC (g)	1961.4	398.3	2161.3	273.5	2488.2	367.9
Total mass (g)	59,230.3	14,066.3	54,366.8	5586.9	71,689.7	12,912.8
RFM (%)	33.0	8.0	22.8	3.1	19.7	3.9
FM/LM ratio (n)	0.5	0.2	0.3	0.1	0.3	0.1
ALMI (kg/m^2^)	5.9	1.4	6.3	0.6	7.7	0.9
**Regional body composition**						
Arms FM (g)	2364.8	1234.8	1243.3	309.7	1516.0	564.4
Arms LM (g)	4068.7	793.5	3868.0	480.0	7009.2	1617.6
Arms BMC (g)	268.0	62.4	263.6	35.0	377.5	115.3
Arms total mass (g)	6703.5	1697.2	5374.9	637.7	8906.8	2018.6
Arms RFM (%)	33.8	11.3	23.0	4.8	17.1	4.6
Legs FM (g)	8278.8	3659.1	5703.1	1069.2	4725.8	1565.5
Legs LM (g)	11,337.7	3733.3	13,263.2	1685.7	16,834.5	3991.6
Legs BMC (g)	608.3	236.0	759.0	110.9	840.5	270.6
Legs total mass (g)	20,224.7	6532.0	19,725.3	2195.3	22,400.8	5368.6
Legs RFM (%)	40.3	10.3	28.9	4.4	20.9	4.3
Trunk FM (g)	8181.8	4343.8	4659.7	1099.6	7323.1	3876.1
Trunk LM (g)	19,309.5	2668.4	19,853.7	2275.1	27,413.4	3624.4
Trunk BMC (g)	516.4	103.1	548.1	105.3	667.6	128.5
Trunk total mass (g)	28,007.7	6600.6	25,061.5	2971.6	35,404.1	7130.7
Trunk RFM (%)	27.6	8.6	18.5	3.3	19.7	5.7

Legend: F_PI, female athletes with a physical impairment; F_ND, able-bodied female athletes; M_PI, male athletes with a physical impairment; SD, standard deviation; FM, absolute fat mass; LM, lean mass; BMC, bone mineral content; RFM, relative fat mass; FM/LM ratio, fat mass to lean mass ratio; ALMI, appendicular lean mass index.

**Table 2:** Results of the Independent Samples *t*-Test.

	F_PI vs. F_ND			F_PI vs. M_PI		
	t Value	*p* Value	Effect Size	t Value	*p* Value	Effect Size
**General characteristics**						
Age (years)	0.037	0.971	0.01	−1.008	0.320	0.2
Weight (kg)	1.382	0.175	0.4	−3.341	**0.002**	0.9
Stature (cm)	−1.327	0.192	0.4	−5.319	**<0.001**	1.4
BMI (kg/m^2^)	2.103	**0.042**	0.7	−0.695	0.491	0.2
**Whole-body analysis**						
FM (g)	3.650	**0.001**	1.2	2.059	**0.047**	0.7
LM	−1.244	0.221	0.4	−8.287	**<0.001**	2.2
BMC (g)	−1.850	0.072	0.6	−4.831	**<0.001**	1.3
Total mass (g)	1.437	0.159	0.5	−3.249	**0.002**	0.9
RFM (%)	4.820	**<0.001**	1.5	5.969	**<0.001**	1.8
FM/LM ratio (n)	4.707	**<0.001**	1.5	5.659	**<0.001**	1.7
ALMI (kg/m^2^)	−1.312	0.197	0.4	−5.212	**<0.001**	1.5
**Regional analysis**						
Arms FM (g)	3.940	**<0.001**	1.2	2.691	**0.011**	0.8
Arms LM (g)	0.968	0.339	0.3	−7.660	**<0.001**	2.0
Arms BMC (g)	0.270	0.788	0.1	−3.963	**<0.001**	1.0
Arms total mass (g)	3.277	**0.002**	1.0	−3.940	**<0.001**	1.0
Arms RFM (%)	3.923	**<0.001**	1.2	6.038	**<0.001**	1.7
Legs FM (g)	3.022	**0.004**	1.0	3.809	**0.001**	1.1
Legs LM (g)	−2.102	**0.042**	0.7	−4.769	**<0.001**	1.2
Legs BMC (g)	−2.585	**0.014**	0.8	−3.149	**0.003**	0.8
Legs total mass (g)	0.324	0.748	0.1	−1.366	0.180	0.3
Legs RFM (%)	4.525	**<0.001**	1.4	7.512	**<0.001**	2.1
Trunk FM (g)	3.515	**0.001**	1.1	0.491	0.626	0.2
Trunk LM (g)	−0.694	0.492	0.2	−9.131	**<0.001**	2.2
Trunk BMC (g)	−0.961	0.343	0.3	−4.495	**<0.001**	1.1
Trunk total mass (g)	1.820	0.077	0.6	−3.781	**0.001**	0.9
Trunk RFM (%)	4.443	**<0.001**	1.4	3.241	**0.003**	0.9

Legend: F_PI, female athletes with a physical impairment; F_ND, able-bodied female athletes; M_PI, male athletes with a physical impairment; vs., versus; FM, absolute fat mass; LM, lean mass; BMC, bone mineral content; RFM, relative fat mass; FM/LM ratio, fat mass to lean mass ratio; ALMI, appendicular lean mass index. Statistically significant results are in bold.

## Data Availability

Research data will be made available upon request to the corresponding author.
